# Supplementary figures and images for: Coverage and effectiveness of conditional cash transfer for people with drug resistant tuberculosis in Zimbabwe: A mixed methods study
Source: PLOS Glob Public Health. 2022 Dec 21;2(12):e0001027. doi: 10.1371/journal.pgph.0001027 (PMC10021731; doi:10.1371/journal.pgph.0001027)

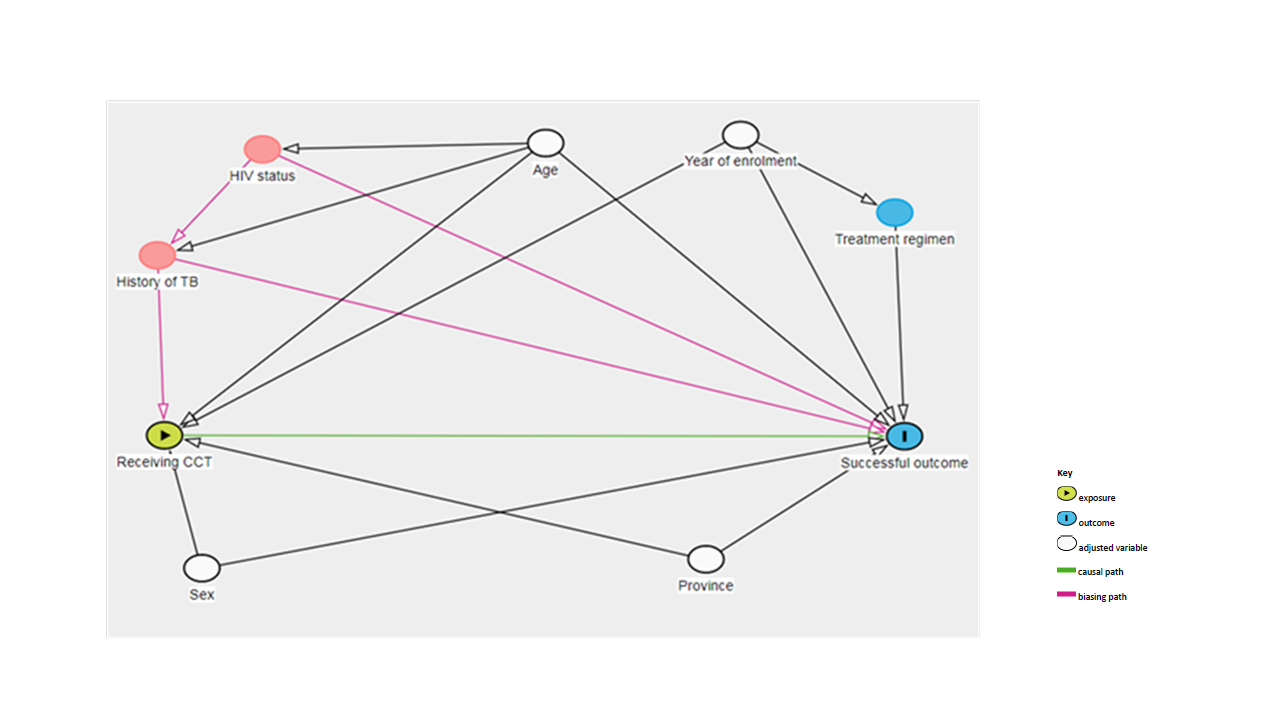

Supplement: S1 Fig — (TIF) [file pgph.0001027.s001.tif]

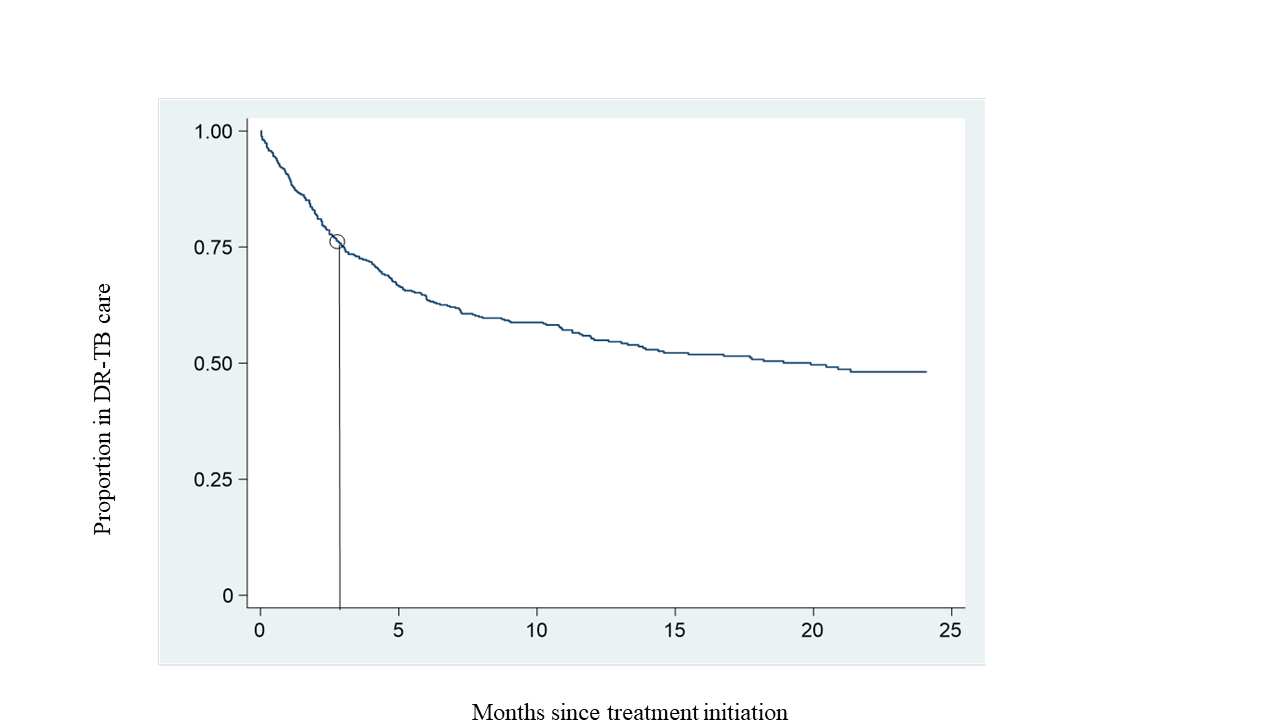

Supplement: S2 Fig — (TIF) [file pgph.0001027.s002.tif]
