# Supplementary material for: Coverage and effectiveness of conditional cash transfer for people with drug resistant tuberculosis in Zimbabwe: A mixed methods study
Source: PLOS Glob Public Health. 2022 Dec 21;2(12):e0001027. doi: 10.1371/journal.pgph.0001027 (PMC10021731; doi:10.1371/journal.pgph.0001027)
Supplement: S1 Table — (DOCX) [file pgph.0001027.s003.docx]

**S1 Table:** Demographic and clinical characteristics of DR-TB participants who were enrolled in the qualitative interviews, Zimbabwe, 2020-2021

| Study ID | HIV  status | Duration of treatment | Marital status | History of TB | Formally employed | Received CCT |
| --- | --- | --- | --- | --- | --- | --- |
| Agnella_F**‡** | Positive | 18-24mo | Single mother (2 children) | No | No | Yes |
| Ruth_F | Positive | 9-12mo | Single mother | Yes. | No | Yes |
| Felistus_F | Negative | 18-24mo | Single, never married | Yes | No | Yes |
| Moses_M | Negative | 9-12mo | Married | No | No | Yes |
| Peter_M | Positive | 18-24mo | Single | No | No | No |
| Caleb_M | Positive | 9-12mo | Married | No | No | Yes |
| Talent_F | Positive | Positive | Single mother | No | No | No |
| Lydia_F | Positive | 18-24mo | Single, never married | No | Yes | Yes |
| Godfrey_M | Positive | 18-24mo | Single, never married | Yes | No | Yes |
| Sipho_M | Positive | 18-24mo | Married | Yes | No | Yes |
| Spiwe_F | Negative | 9-12mo | Married | No | No | No |
| Loveness_F | Negative | 18-24mo | Single mother (1 child) | No | No | Yes |
| Tecla_F | Positive | 18-24mo | Single mother (1 child) | No | No | Yes |
| Nyenge_M | Positive | 9-12mo | Single | Yes | No | Yes |
| Wembe_M | Positive | 9-12mo | Married | No | No | Yes |
| Fanuel_M | Positive | 9-12mo | Married with 2 children | No | No | Yes |

‡=pseudonym and sex of participant; CCT=Conditional cash transfer
